# Chinese Proprietary Medicine Xianling Gubao Capsule for Osteoporosis: A Systematic Review and Meta-Analysis of Randomized Clinical Trials

**DOI:** 10.3389/fendo.2022.870277

**Published:** 2022-04-07

**Authors:** Bai-Ru Cheng, Rou-Yan Wu, Qin-Yang Gao, Kai-Xin Jiang, Shuang-Sang Li, Shi-Hao Qi, Ming-Yi Yuan, Jian-Ping Liu

**Affiliations:** ^1^ The First School of Clinical Medicine (Dongzhimen Hospital), Beijing University of Chinese Medicine, Beijing, China; ^2^ The Second School of Clinical Medicine (Dongfang Hospital), Beijing University of Chinese Medicine, Beijing, China; ^3^ Centre for Evidence-Based Chinese Medicine, Beijing University of Chinese Medicine, Beijing, China

**Keywords:** Xianling Gubao capsule, Chinese proprietary medicine, osteoporosis, quality of life, bone mineral density, systematic review, meta-analysis

## Abstract

**Objective:**

To assess the benefit and harm of Chinese medicine Xianling Gubao (XLGB) capsule compared to conventional medication or placebo to inform clinical practice.

**Methods:**

We included randomized controlled trials (RCTs) with Jadad score ≥3 of XLGB capsule compared to pharmaceutical medication, placebo, or no treatment for primary osteoporosis. We conducted searches in EMBASE, Cochrane CENTRAL, MEDLINE, China National Knowledge Infrastructure, VIP, Wanfang, and Chinese Biomedical Literature Database (Sino-Med) from their inception till November 13^th^, 2021. Study selection and data extraction were done by two authors independently. The methodological quality of the RCTs was assessed using Cochrane’s risk of bias tool. The effect size was presented as risk ratio (RR) or mean difference (MD) with their 95% confidence interval (CI).

**Results:**

Our searches identified 2292 records and after exclusions, eight trials involving 846 participants were included. There was no statistically significant difference between conventional medications with or without XLGB on new fracture (RR: 0.50, 95% CI: [0.13, 1.87]). Quality of life by SF-36 questionnaire of XLGB plus calcium carbonate, vitamin D_3_, and calcitriol was improved than that of without XLGB (MD: 6.72 scores, 95% CI: [2.82, 10.62]). XLGB increased bone mineral density similarly as calcium carbonate plus vitamin D_3_ (MD: 0.21, 95% CI: [-0.16, 0.58]) or as alendronate sodium, calcium carbonate plus vitamin D_3_ (MD: 0.00, 95% CI: [-0.10, 0.10]), but it had no additional effect as an add-on treatment to conventional medications (MD: 0.13, 95% CI: [-0.12, 0.37]). XLGB relieved pain *via* visual analog scale more effectively when combined with medications (MD: -1.55 score, 95% CI: [-2.47, -0.63]). XLGB as monotherapy did not increase adverse events (RR: 0.63, 95% CI: [0.28, 1.41]), or as an add-on treatment (RR: 0.25, 95% CI: [0.03, 2.16]).

**Conclusion:**

This systematic review shows that XLGB capsule appears to be safe and has a beneficial effect on the quality of life and pain relief when used alone or in combination with conventional medications in osteoporosis patients. Further large, rigorous trials are warranted to test its long-term benefit.

## 1 Introduction

Osteoporosis is characterized by the deformation of bone’s microarchitecture and fragility of bones, resulting in the increment of fractures, especially in postmenopausal women. Its diagnosis criteria vary (1), resulting in a wide range of reported incidence, fractures are a great threat for osteoporosis patients since they not only cause pain or humpback but also impair dignity and quality of life. Therefore, reducing fractures has become the primary goal in osteoporosis control (2).

Current therapies include physical activity, nutrient supplements, antiresorptive drugs, and anabolic drugs, while pharmacologic treatments are the most recommended (3, 4). Exercise and balance programs such as Tai-Chi have been proved to improve coordination, reducing falling-downs, consequently lowering fracture rates (5). Lifestyle changes such as quitting cigarette smoking or reducing alcohol intake help increase bone mineral density (BMD) and reduce the risk of falls (6). Nutrient supplements calcium and vitamin D have a controversial effect preventing fractures (7), and it was found to increase cardiovascular events, especially myocardial infarction (8). Pharmacologic agents such as alendronate, zoledronic acid, and calcitonin aim at preventing bone resorption or stimulating bone formation and have been shown to improve BMD and reduce the risk of fractures. The recommended therapy for women with low BMD and a fracture risk or history includes all the means mentioned above, but in China, some patients would also seek help from traditional Chinese medicine (TCM).

Xianling Gubao (XLGB) capsule is the most recommended Chinese proprietary medicine for osteoporosis treatment. It was approved by the China Food and Drug Administration in 2002 (9). It contains *Longspur epimedium* (Yin Yang Huo), *Radix dipsaci* (Xuduan), *Salvia miltiorrhiza* (Danshen), *Rhizoma anemarrhenae* (Zhimu), *Rehmannia glutinosa* (Dihuang), and *Psoralea corylifolia* (Bu Gu Zhi). Based on network pharmacology and molecular docking, the identified components such as icariin, quercetin, and luteolin, may aim at WnT, TNF, MAPK, PI3K-Akt pathways, relating to targets including STAT3, MAPK14, JUN, IL-2, and EGFR, which are important in bone homeostasis (10). XLGB capsule was found to downregulate RANKL mRNA and upregulate osteoprotegerin mRNA, which combines with RANKL to reduce osteoclasts, finally inhibiting bone destruction (11). It was also reported that the combination of six typical absorbed constituents of XLGB capsule could promote MC3T3-E1 cells’ differentiation and mineralization (12). In TCM theory, most bone diseases pertain to the deficiency of the kidney and the blood stasis in meridians which causes pain, thus, this formula could nourish the liver and the kidney, promote blood circulation, and remove meridian obstruction, to strengthen the muscles and bones. There is an increasing number of clinical trials on XLGB capsule in China (13–16). Although most trials present positive findings, some of them are of low quality, lacking blinding or proper randomization methods. Therefore, we thought it necessary to do a systematic review of randomized trials with adequate quality to provide reliable evidence for the clinical use of XLGB capsule.

## 2 Methods

### 2.1 Search Strategy

We retrieved publications using computerized searches by EMBASE, Cochrane CENTRAL, MEDLINE, China National Knowledge Infrastructure, Wanfang, VIP, and Chinese Biomedical Literature Database (Sino-Med), with no limit on inception date or language. The last search date was November 13^th^, 2021. The search strategy for MEDLINE (*via* PubMed) is listed in **Appendix 1**.

### 2.2 Inclusion Criteria

Study design: parallel-group, randomized clinical trials regardless of blinding in all languages. There was no limit on the number of participants. Studies with a Jadad score ≥3 were included [using the Jadad scale (17)].

Patients with primary osteoporosis, diagnosed according to any clearly defined criteria were included, regardless of age, gender, or ethnic origin. Those whose chief complaint was a recent fracture and those with secondary osteoporosis such as diabetes-induced, rheumatoid arthritis-induced, or corticosteroid-induced osteoporosis were excluded.

Intervention: Chinese proprietary medicine Xianling Gubao capsule with a minimum treatment duration of three months.

Control intervention could be no treatment, placebo, or conventional pharmaceutical medicine (such as alendronate, zoledronic acid, hormone replacement therapy, bisphosphonate, calcitonin, calcium, and vitamin D).

Co-intervention was allowed as long as the Xianling Gubao group received the same conventional pharmaceutical medicine as at least one comparison group and the only difference between them was the add-on Xianling Gubao capsule.

### 2.3 Outcome Measures

#### 2.3.1 Primary Outcomes

1. Number of individuals with new fractures; 2. Quality of life (QoL) is measured by a validated tool or scale.

#### 2.3.2 Secondary Outcomes

1. Bone mineral density (BMD); detected by one of the following methods of examination: single-photon absorptiometry (SPA), dual photon absorptiometry (DPA), quantitative computed tomography (QCT), dual-energy X-ray absorptiometry (DXA), or peripheral dual-energy X-ray absorptiometry (pDXA); 2. Biochemical indicators: serum calcium (Ca), phosphorus (P), bone alkaline phosphatase (BALP), OC (osteocalcin), TRACP (tartrate-resistant acid phosphatase); 3. Pain, muscle fatigue, and limited mobility; 4. Number and types of adverse events.

### 2.4 Study Selection

Two authors (BR Cheng and RY Wu) independently screened the titles and abstracts of all records. We retrieved the full texts of potentially eligible studies for further identification. Any uncertainty or discrepancy was resolved by discussion with a third author (JP Liu).

### 2.5 Data Extraction

Two authors (MY Yuan and SS Li) independently extracted data using a predesigned data form using Excel (version Microsoft Excel 2016). Extracted data were checked together, and any disagreements were resolved by discussion with SH Qi.

### 2.6 Risk of Bias Assessment

Since only RCTs were included, the risk of bias was assessed through Cochrane’s risk of bias Tool for Randomized Trials (RoB) (18). Two authors (QY Gao and KX Jiang) independently assessed the risk of bias. Disagreements were resolved by discussion with a third author (BR Cheng). The risk of bias was assessed through the following five domains: 1) bias arising from the randomization process; 2) bias due to deviations from intended interventions; 3) bias due to missing outcome data; 4) bias in the measurement of the outcome; 5) bias in the selection of the reported result.

### 2.7 Data Analysis and Synthesis

We provided a narrative synthesis of the findings from the included studies and worked with the data within a meta-analysis, through Review Manager 5.3. Heterogeneity related to the results of the studies was assessed using both the chi-square test and the I² statistic. If data had been sufficiently homogenous (I^2^ <50%), we would pool the results using a fixed-effect model, with a mean difference (MD) for continuous outcomes (in our review referring to QoL, BMD, Ca, P, BALP, OC, TRACP, and VAS score) and risk ratio (RR) for dichotomous outcomes (referring to the number of individuals with new fractures and adverse events), and calculated 95% confidence interval (CI) and two-sided *p* values for each outcome. We provided summaries of effect estimates for each study by calculating RR or MD. We considered an I² value greater than 50% as high heterogeneity. If there had been high heterogeneity across included studies, we would use a random-effect model or only provide a narrative synthesis of the findings.

## 3 Results

### 3.1 Study Selection

**Figure 1:**
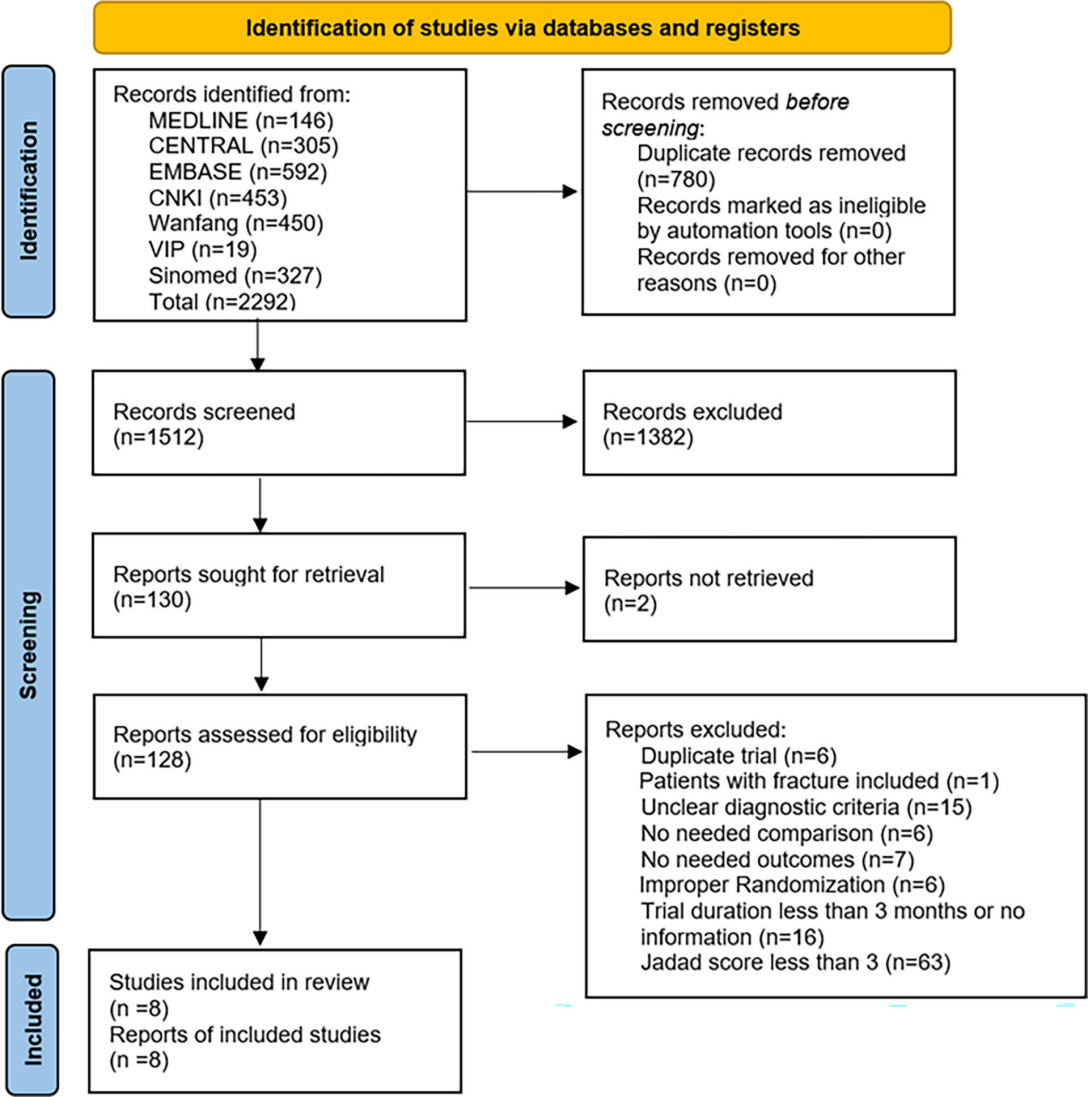
Flowchart of the study selection process.

Database searches initially identified 2292 records published in English or Chinese. The last search date was November 13^th^, 2021. After removing duplications, 1512 articles were screened by their titles and abstracts. 130 reports were sought for retrieval of full text but we failed to find two of them, as they had no available resources online, and 128 out of 130 reports were assessed for eligibility. A flowchart (**Figure 1**) with the number of included studies at each step was established, including reasons for excluding studies. Eight trials were finally included in the qualitative and quantitative synthesis.

### 3.2 Study Characteristics

**Table 1** presents the characteristics of the included studies, and five out of the eight studies were master’s or doctor’s thesis, and three were published studies in journals. The included eight studies involved 846 participants (ranging from 60 to 180). The conventional treatments in the control groups included calcium carbonate and vitamin D_3_, calcitriol, alendronate sodium, and carbocalcitonin, as well as the combination of some of them. The treatment duration ranged from three to twelve months.

**Table 1:** Characteristics of randomized controlled trials on Xianling Gubao capsule for osteoporosis.

Study	Condition	Sample Size (Experiment/control)	Experiment	Comparators	Duration (months)	Jadad Score (points out of 5)
**Feng et al.** (19)	Senile osteoporosis	30/30	Calcium carbonate and vitamin D_3_ 600mg Qd	Calcium carbonate and vitamin D_3_ 600mg Qd	12	3
			Calcitriol 0.25μg Qd	Calcitriol 0.25μg Qd		
			Xianling Gubao capsule 1.5g Tid			
**Li** (20)	Senile osteoporosis	30/30	Calcium carbonate and vitamin D_3_ 600mg Bid	Calcium carbonate and vitamin D_3_ 600mg Bid	3	3
			Xianling Gubao capsule 1.5g Tid			
**Liu and Bai** (21)	Senile osteoporosis	76/74	Alendronate sodium 70mg Qw	Alendronate sodium 70mg Qw	6	3
			Calcium carbonate 500mg Qd	Calcium carbonate 500mg Qd		
			Xianling Gubao capsule 1.0g Bid			
**Ouyang** (22)	Postmenopausal osteoporosis	30/30	Calcium carbonate and vitamin D_3_ 600mg Qd	Calcium carbonate and vitamin D_3_ 600mg Qd	3	3
			Xianling Gubao capsule 1.5g Bid			
**Xu** (23)	Senile osteoporosis	42/42	Carbocalcitonin 10IU Biw*	Carbocalcitonin 10IU Biw*	6	3
			Calcium carbonate and vitamin D_3_ 600mg Bid	Calcium carbonate and vitamin D_3_ 600mg Bid		
			Calcitriol 0.25μg Bid	Calcitriol 0.25μg Bid		
			Xianling Gubao capsule 1.5g Bid			
**You** (24)	Postmenopausal osteoporosis	20/20	Xianling Gubao capsule 1.5g Bid	Alendronate sodium 70mg Qw	6	3
				Calcium carbonate and vitamin D_3_ 600mg Qd		
**Zhang** (25)	Senile osteoporosis	30/30	Xianling Gubao capsule 1.5g Bid	Calcium carbonate and vitamin D_3_ 600mg Bid	3	3
**Zhu et al.** (26)**^#^**	Postmenopausal osteoporosis	61/61	Calcium carbonate 500mg Qd	Calcium carbonate 500mg Qd	12	4
			Vitamin D_3_ 200IU Qd	Vitamin D_3_ 200IU Qd		
			Xianling Gubao capsule 3g/day			
**Zhu et al.** (26)**^#^**	Postmenopausal osteoporosis	58/61	Calcium carbonate 500mg Qd	Calcium carbonate 500mg Qd	12	4
			Vitamin D_3_ 200IU Qd	Vitamin D_3_ 200IU Qd		
			Xianling Gubao capsule 6g/day			

Qd, once daily; Bid, twice daily; Tid, three times daily; g, gram; mg, milligram; μg, microgram; Qw, once per week; Biw, twice per week.

*intramuscular-injection; ^#^Zhu 2012 contains 2 intervention groups and 1 control group.

### 3.3 Risk of Bias of Individual Studies

**Figure 2:**
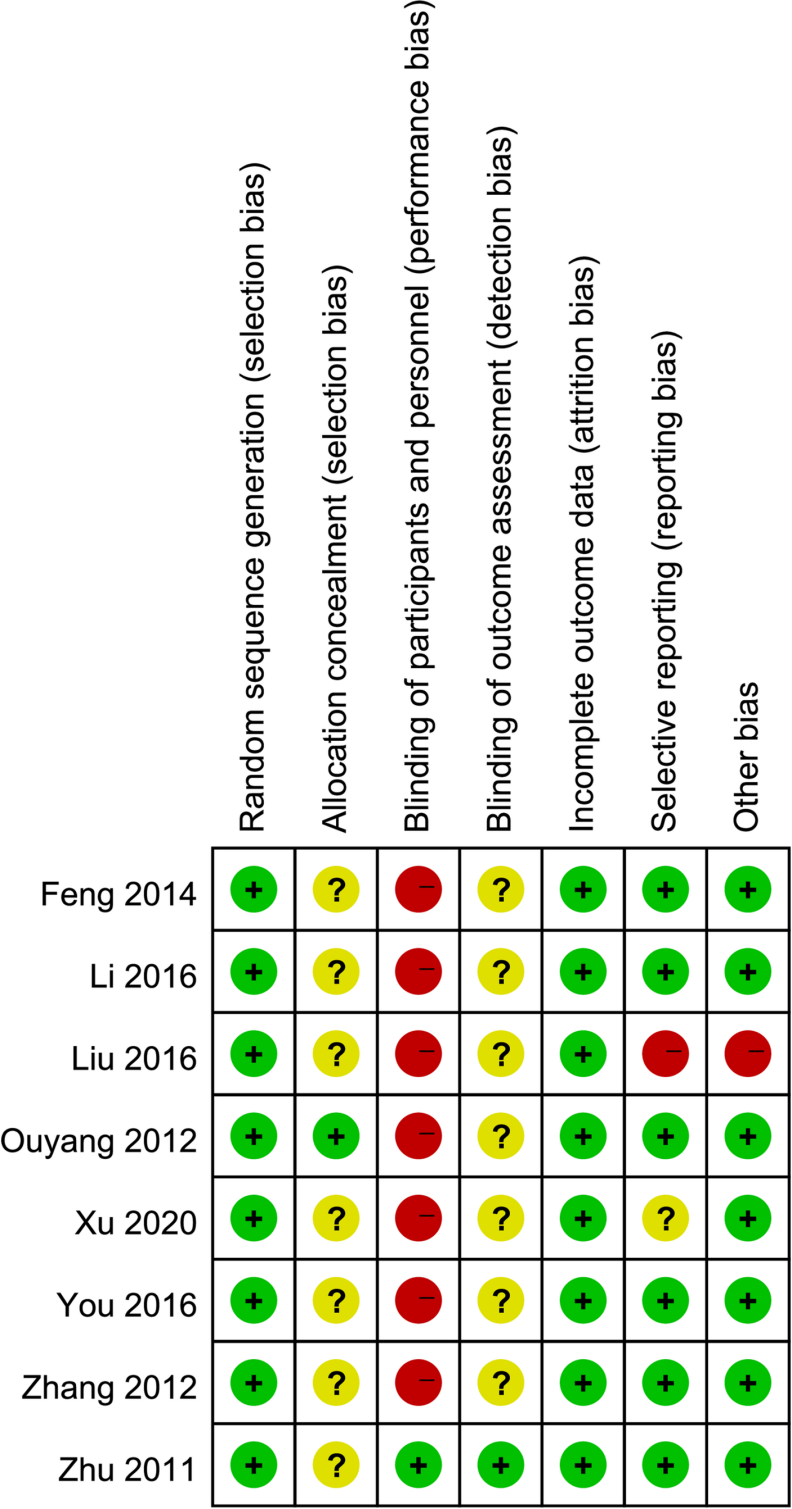
Risk of bias summary: a review of authors’ judgments about each risk of bias item for each included study.

**Figure 3:**
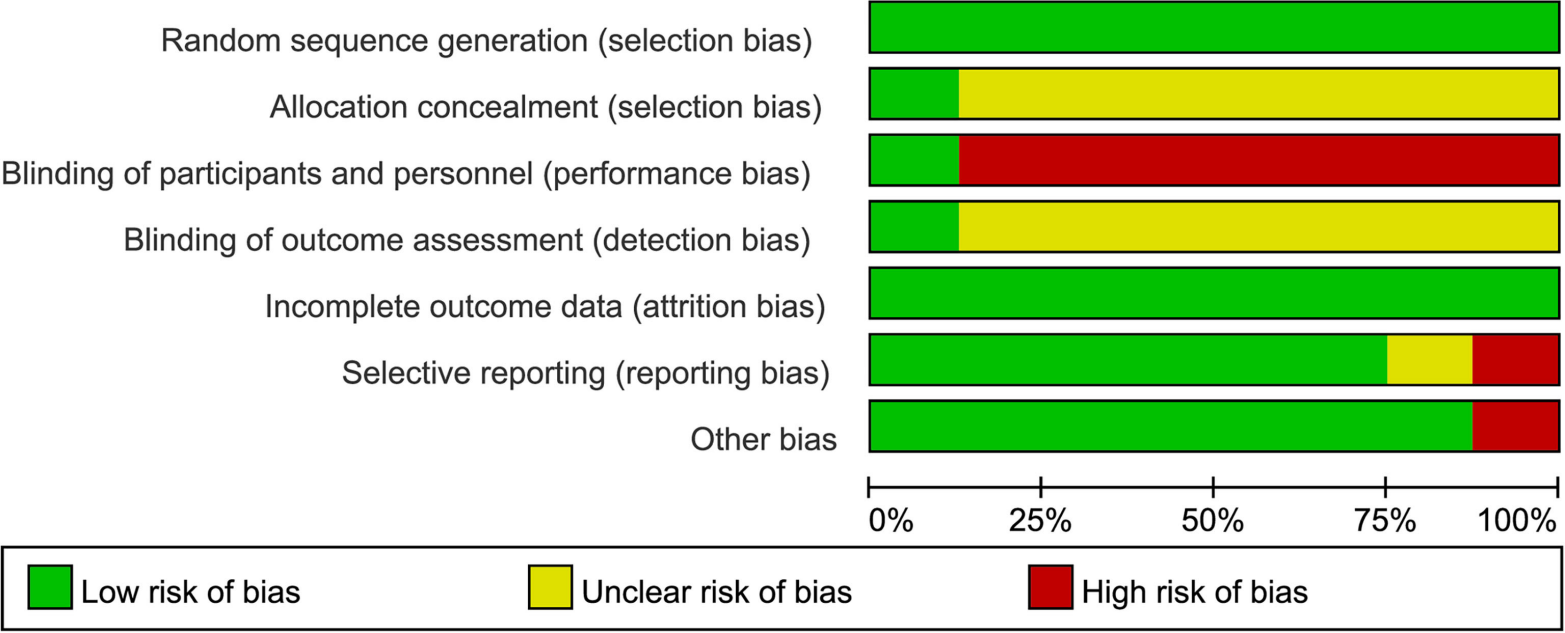
Risk of bias graph: A review of authors’ judgments about each risk of bias item presented as percentages across all included studies.

**Figures 2**, **3** are summaries of each included studies’ risk of bias. Overall, all included studies were of unclear or high risks, which was mostly contributed by lack of randomization sequence concealment and absence of blinding process. All studies had a proper randomization process, but none reported blinding of participants, personnel, or outcome assessment except 1 triple-blinded study, using a placebo (26). Only one study reported randomization concealment using sealed envelopes (22). Subjective outcomes such as the VAS score and the QoL score could have been biased due to the lack of participant blinding, but objective outcomes like bioindicator levels are less likely to be biased.

### 3.4 Primary Outcomes

#### 3.4.1 Number of Individuals With New Fractures

Only one trial reported this outcome (23). In the twelve-month follow-up after the six-month treatment, there was no significant difference between XLGB plus medications (carbocalcitonin, calcium carbonate, vitamin D_3_, and calcitriol) and medications on reported new fracture onset (3/42 versus 6/42; RR: 0.50, 95% CI: [0.13, 1.87]).

#### 3.4.2 Quality of Life (QoL)

Only one trial used the SF-36 health survey questionnaire (eight domains with total scores of 100) to assess patients’ quality of life (17). It showed a significant difference between calcium carbonate, vitamin D_3_, and calcitriol with or without XLGB (MD: 6.72 scores, 95% CI: [2.82, 10.62]) (19).

### 3.5 Secondary Outcomes

#### 3.5.1 Bone Mineral Density (BMD)

Five trials reported BMD of the lumbar spine. Two reported BMD in T scores and one did not mention the place where the BMD was detected (21), making its result unable to be pooled. In two comparisons where the units were g/cm^2^ and T score, XLGB increased bone mineral density to a similar extent as calcium carbonate plus vitamin D_3_ (MD: 0.21, 95% CI: [-0.16, 0.58]), or as alendronate sodium, calcium carbonate plus vitamin D_3_ (MD: 0.00, 95% CI: [-0.10, 0.10]), but it had no additional effect as an add-on to conventional medications (MD: 0.13, 95% CI: [-0.12, 0.37]), though differences were seen another comparison (T score) of carbocalcitonin, calcium carbonate, vitamin D_3_, and calcitriol with or without XLGB (MD: 0.11, 95% CI: [0.09, 0.13]).

#### 3.5.2 Biochemical Indicators

##### 3.5.2.1 Serum Calcium and Phosphorus Levels

No comparison showed a difference between XLGB plus conventional treatment and the conventional treatment, but in one study (25), serum calcium and phosphorus levels in XLGB group were significantly lower than calcium carbonate plus vitamin D_3_ group (**Table 2**).

**Table 2:** Mean differences of continuous outcomes.

	Study ID	MD [95% CI]	*P* value
***Bone mineral density (lumbar spine, g/cm^2^)***			
**XLGB plus conventional versus conventional treatment**	Feng et al. (19)	0.06 [-0.00, 0.12]	0.06
	Li (20)	0.04 [0.02, 0.06]	0.0001
	Ouyang (22)	0.54 [0.51, 0.56]	<0.00001
	Zhu et al. (26)	0.00 [-0.04, 0.04]	1.00
	Zhu et al. (26)	0.00 [-0.03, 0.03]	1.00
	Pooled result	0.13 [-0.12, 0.37]	0.31
**XLGB versus calcium carbonate and vitamin D_3_**	Zhang (25)	0.03 [-0.02, 0.08]	0.17
***Bone mineral density (T score)***			
**XLGB plus carbocalcitonin, calcium carbonate, vitamin D_3_, and calcitriol versus carbocalcitonin, calcium carbonate, vitamin D_3_, and calcitriol**	Xu (23)	0.11 [0.09, 0.13]	<0.00001
**XLGB versus alendronate sodium, calcium carbonate and vitamin D_3_**	You (24)	-0.05 [-0.25, 0.15]	0.63
	You (24)	0.02 [-0.09, 0.13]	0.73
	Pooled result	0.00 [-0.10, 0.10]	0.56
***Serum calcium (Ca, mmol/L)***			
**XLGB plus conventional versus conventional treatment**	Feng et al. (19)	0.02 [-0.10, 0.14]	0.73
	Ouyang (22)	-0.20 [-0.33, -0.07]	0.002
	Pooled result	-0.09 [-0.30, 0.13]	0.01
**XLGB versus calcium carbonate and vitamin D_3_**	Zhang (25)	-0.24 [-0.29, -0.19]	<0.00001
***Phosphorus (P, mmol/L)***			
**XLGB plus conventional versus conventional treatment**	Feng et al. (19)	-0.01 [-0.12, 0.10]	0.86
	Ouyang (22)	-0.09 [-0.28, 0.10]	0.35
	Pooled result	-0.03 [-0.13, 0.07]	0.48
**XLGB versus calcium carbonate and vitamin D_3_**	Zhang (25)	-0.24 [-0.34, -0.14]	<0.00001
***Bone alkaline phosphatase (BALP, U/L)***			
**XLGB plus calcium carbonate and vitamin D_3_ versus calcium carbonate and vitamin D_3_**	Ouyang (22)	6.67 [1.88, 11.46]	0.006
**XLGB versus alendronate sodium, calcium carbonate and vitamin D_3_**	You (24)	6.79 [-23.99, 37.57]	0.67
***Osteocalcin (OC, μg/L)***			
**XLGB plus conventional versus conventional treatment**	Li (20)	0.47 [0.41, 0.53]	<0.00001
	Xu (23)	0.12 [0.09, 0.15]	<0.00001
	Zhu et al. (26)	0.45 [-2.50, 3.40]	0.76
	Zhu et al. (26)	0.76 [-2.32, 3.84]	0.63
	Pooled result	0.30 [-0.03, 0.64]	0.08
**XLGB versus calcium carbonate and vitamin D_3_**	Zhang (25)	0.37 [0.33, 0.42]	<0.00001
***Tartrate-resistant acid phosphatase 5b (TRACP5b, nmol/L)***			
**XLGB plus conventional versus conventional treatment**	Feng et al. (19)	-0.95 [-1.48, -0.42]	0.0004
	Ouyang (22)	-0.49 [-0.83, -0.15]	0.005
	Pooled result	-0.67 [-1.11, -0.23]	0.003
**XLGB versus alendronate sodium, calcium carbonate and vitamin D_3_**	You (24)	0.36 [0.10, 0.62]	0.007
**Pain (VAS score)**			
**XLGB plus conventional versus conventional treatment**	Feng et al. (19)	-0.99 [-1.38, -0.60]	<0.00001
	Li (20)	-1.16 [-1.78, -0.54]	0.0002
	Liu and Bai (21)	-2.88 [-3.19, -2.57]	<0.00001
	Ouyang (22)	-1.94 [-2.48, -1.40]	<0.00001
	Xu (23)	-0.79 [-1.02, -0.56]	<0.00001
	Pooled result	-1.55 [-2.47, -0.63]	0.0009
**XLGB versus alendronate sodium, calcium carbonate and vitamin D_3_**	You (24)	-0.12 [-0.82, 0.58]	0.74

XLGB, Xianling Gubao capsule; MD, Mean difference; CI, Confidence interval; VAS, Visual analogue scale.

*lumbar spine; ^#^femoral neck.

##### 3.5.2.2 BALP, Osteocalcin, and TRACP5b Levels

XLGB plus calcium carbonate and vitamin D_3_ resulted in a higher level of BALP than that of calcium carbonate and vitamin D_3_ (MD: 6.67 U/L, 95% CI: [1.88, 11.46]), but XLGB alone was no better than calcium carbonate plus vitamin D_3_ (MD: 6.79 U/L, 95% CI: [-23.99, 37.57]).

A higher osteocalcin level was observed in XLGB group compared to the calcium carbonate and vitamin D_3_ group. The combination of the two treatments showed no differences in osteocalcin than calcium carbonate and vitamin D_3_ alone (**Table 2**).

The results of the TRACP5b level were also conflicting. It was decreased in XLGB plus conventional treatment group compared to calcium carbonate, vitamin D_3_, plus calcitriol group but was higher in XLGB group compared to alendronate sodium plus calcium carbonate and vitamin D_3_ (**Table 2**).

#### 3.5.3 Pain, Muscle Fatigue, and Limited Mobility

No trials reported fatigue or limited mobility. VAS score (0-10 scale) was used by all studies to measure pain. XLGB plus conventional medications significantly relieved pain than conventional medications (MD: -1.55, 95% CI: [-2.47, -0.63]). Compared to alendronate sodium plus calcium carbonate and vitamin D_3_, XLGB alone did not show significant difference (MD: -0.12, 95% CI: [-0.82, 0.58]).

#### 3.5.4 Number and Types of Adverse Events

There was no significant difference in the overall rate of any adverse events between XLGB (both used alone and as add-on treatment) and conventional medication. No severe adverse events were reported. The reported adverse events in XLGB group included one case with headache, one losing appetite, one red flush, five gastrointestinal reactions, and one constipation. (**Table 3**).

**Table 3:** Risk ratio of any adverse events.

	Study ID	RR [95% CI]	*P* value
***Any adverse events***			
**XLGB plus conventional versus conventional treatment**	Feng et al. (19)	1.00 [0.22, 4.56]	1.00
	Liu and Bai (21)	0.61 [0.21, 1.78]	0.82
	Xu (23)	0.33 [0.04, 3.08]	0.76
	Zhu (26)	1.19 [0.61, 2.33]	0.61
	Pooled result	0.90 [0.54, 1.50]	0.69
**XLGB versus conventional treatment**	You (24)	0.33 [0.01, 7.72]	0.82
	Zhang (25)	0.20 [0.01, 4.00]	0.58
	Pooled result	0.25 [0.03, 2.16]	0.59

XLGB, Xianling Gubao capsule; RR, Risk ratio; CI, Confidence interval.

### 3.6 Summary of the Evidence

Due to the small number of included trials, for primary outcomes–number of new fractures and quality of life–the results were not pooled, and XLGB did not significantly reduce fractures but improved quality of life. **Table 2** presents the full results of all secondary outcomes. For bone mineral density, the direct comparison of XLGB to conventional treatments had insufficient data, but the effect of XLGB as an add-on treatment was not significant. For calcium, phosphorus, and osteocalcin levels, pooled results showed no differences in the conventional treatment with or without XLGB. It was shown that XLGB significantly reduced TRACP5b levels and VAS score, indicating that it could inhibit bone deformation and relieve pain. **Table 3** shows safety outcomes, where XLGB did not result in more adverse events when added to conventional medications or compared to them.

## 4 Discussion

This systematic review and meta-analysis investigated the efficacy and safety of XLGB capsule in patients with primary osteoporosis. Overall, XLGB capsule is a safe treatment that improved quality of life and reduces pain as an add-on treatment compared to conventional medications, including calcium carbonate, vitamin D_3_, calcitriol, alendronate sodium, and carbocalcitonin, or the combination of some of them, and XLGB plus conventional treatments had a better effect than conventional medications alone. Its effects on new fractures, bone mineral density, serum calcium, and serum phosphorous levels were not significant. The results of BALP, osteocalcin, and TRACP5b levels were controversial, which could be a result of insufficient data.

XLGB capsule is a Chinese proprietary medicine made of six herbs, making its components difficult to be fully analyzed. Based on network pharmacology and molecular docking, the identified components were cryptotanshinone, chryseriol, kaempferol, anhydroicaritin, quercetin, and luteolin, which might aim at WnT, TNF, MAPK, PI3K-Akt pathways, relating to targets including STAT3, MAPK14, JUN, IL-2, and EGFR (10). Among the mentioned molecules, flavonoids such as quercetin, luteolin, icariin are widely believed to be the main components. Quercetin, kaempferol, and icariin inhibit RANKL activation and OPG/RANK/RANKL is important in osteoclastic differentiation (27, 28). Luteolin stimulates osteoblast proliferation *via* Semaphorin 3A/Neuropilin-1/Plexin A1 pathway (29), and cryptotanshinone improves osteoblastic differentiation and reduces E_2_ to inhibit bone absorption (30, 31). Also, PI3K-Akt, TNF, and MAPK pathways have to do with osteoblastic differentiation, which promotes bone formation (32, 33). Furthermore, by analyzing the absorbed components in rats’ serum after intragastric administration by HPLC-MS/MS analysis, Yao et al. confirmed 15 components including sweroside, epimedin B, isopsoralen, asperosaponin VI, and neobavaisoflavone, which came from four herbs - *Longspur epimedium*, *Radix dipsaci*, *Rhizoma anemarrhenae*, and *Psoralea corylifolia* - in the formula (34). Sweroside interacts with membrane estrogen receptor-α and p38 pathway to promote osteoblastic differentiation and mineralization (35). Epimedin B downregulates PI3K-Akt, MAPK, and PPAR signaling pathways, which are mentioned above to result in bone destruction in mice (36) and have estrogen-like effects (37). Some other compounds like isopsoralen, asperosaponin VI, and neobavaisoflavone have also been proved to either enhance osteogenesis or inhibit osteoclast activation (38–42). Moreover, Wu found that the combination of six typical absorbed constituents of XLGB capsule could promote MC3T3-E1 cells’ differentiation and mineralization, which were not seen in any single-constituent groups, suggesting that there may be some unknown interactive mechanism of the combination (12). Moreover, Ren et al., found that XLGB capsule downregulated RANKL mRNA and upregulated the mRNA of osteoprotegerin, which combines with RANKL to reduce osteoclasts, finally inhibiting bone destruction (11). By analyzing and testing the components of XLGB capsule, we expect new phytomedicines which are more effective and with fewer adverse events.

One previous meta-analysis in Chinese reviewed the efficacy and safety of XLGB capsule in osteoporosis (43). Significant improvement in BMD, VAS, ALP, Ca, and BGP from XLGB was found, but it did not report quality of life or pain, and the difference in BMD was only slightly statistically significant that it may have limited clinical significance. Although the review specified primary osteoporosis, it included different osteoporosis. In addition, the review included randomized trials without any limitation to trial quality or treatment duration. Trials without proper randomization procedures may exaggerate the treatment effect. Compared to this review, our review has some strengths: 1) the first meta-analysis on this topic written in English and only included RCTs with adequate randomization, making the results more reliable; 2) our primary outcomes focusing on new fractures and quality of life, which are important to patients.

However, we do have some limitations: 1) the small number of included trials since we limited our inclusion criteria to those trials with moderate quality (Jadad score ≥3); 2) lack of placebo-controlled, double-blind trial, and add-on trials make blinding to participants or investigators not possible; 3) although we included trials with an adequate generation of allocation sequence, no trial reported allocation concealment, which may cause performance bias in outcome measurement such as VAS and quality of life; 4) small sample sizes (60-180 patients) of included trials may be underpowered for the effect estimates; and 5) the conventional medications used in control groups were diverse, including different combinations, so it was difficult to do subgroup analysis to investigate their efficacy respectively.

Clinicians should be aware that current evidence for XLGB capsule is limited due to small trials or a high risk of bias. Therefore, we suggest more solid evidence before the recommendation of its clinical use. We encourage placebo-controlled, double-blind trials with long follow up to test its efficacy and safety for primary osteoporosis. Only when we are confident of its efficacy would we suggest add-on trials of XLGB to current medications, especially paying attention to clinical outcomes such as the number of new fractures and quality of life.

## 5 Conclusion

This systematic review shows that XLGB capsule appears to be safe and may be used alone or with conventional treatments to improve osteoporosis patients’ quality of life and relieve pain. However, current evidence for its efficacy is limited especially for long-term outcomes such as new fractures.

## Data Availability Statement

The original contributions presented in the study are included in the article/supplementary material. Further inquiries can be directed to the corresponding author.

## Author Contributions

B-RC and J-PL conceived this review topic. B-RC, R-YW, and J-PL drafted the study protocol. B-RC and R-YW did database searches, removed duplications, screened titles, abstracts, and full texts of included papers. K-XJ and Q-YG assessed the risk of bias and extracted data. Outcome data were extracted by M-YY and S-SL, checked, and discussed with S-HQ. Data analyses were done by B-RC and discussed with all other members. Finally, the manuscript was revised by J-PL. All authors contributed to the article and approved the submitted version.

## Funding

This review was supported by the National Natural Science Foundation project (No. 81830115), and J-PL was partially supported by the NCCIH grant (AT001293 with sub-award No. 020468C). The funders have no role in the review design, conduct, interpretation, and writing of the report.

## Conflict of Interest

The authors declare that the research was conducted in the absence of any commercial or financial relationships that could be construed as a potential conflict of interest.

## Publisher’s Note

All claims expressed in this article are solely those of the authors and do not necessarily represent those of their affiliated organizations, or those of the publisher, the editors and the reviewers. Any product that may be evaluated in this article, or claim that may be made by its manufacturer, is not guaranteed or endorsed by the publisher.

## Appendix 1

Search strategy for MEDLINE (*via* PubMed):

1. bone diseases, metabolic[mh]
2. osteoporosis[mh]
3. bone density[all fields]
4. bone loss[all fields]
5. osteomalacia[tw]
6. osteodystrophy[tw]
7. osteopenia[tw]
8. bone mass[tw]
9. densitometry[mh]
10. fractures, bone[mh]
11. #1 OR #2 OR #3 OR #4 OR #5 OR #6 OR #7 OR #8 OR #9 OR #10
12. xianlinggubao[tw]
13. xianling gubao[tw]
14. XLGB[tw]
15. xian-ling-gu-bao[tw]
16. drugs, chinese herbal[mh]
17. herbal medicine[mh]
18. plants, medicinal[mh]
19. medicine, traditional[mh]
20. #12 OR #13 OR #14 OR #15 OR #16 OR #17 OR #18 OR #19
21. randomized controlled trial[pt]
22. controlled clinical trial [pt]
23. randomized [tiab]
24. placebo [tiab]
25. clinical trials as topic [mesh:noexp]
26. randomly [tiab]
27. trial [ti]
28. #21 OR #22 OR #23 OR #24 OR #25 OR #26 OR #27
29. animals [mh] NOT humans [mh]
30. #28 NOT #29
31. #11 AND #20 AND #30
